# Racial, Ethnic, and Sex Diversity in Academic Medical Leadership

**DOI:** 10.1001/jamanetworkopen.2023.35529

**Published:** 2023-09-25

**Authors:** Austin M. Meadows, Madelyn M. Skinner, Alaa A. Hazime, Russell G. Day, Jessi A. Fore, Charles S. Day

**Affiliations:** 1 Henry Ford Health, Detroit, Michigan; 2 Wayne State University School of Medicine, Detroit, Michigan; 3 Michigan State University College of Human Medicine, Grand Rapids

## Abstract

**Importance:**

For the past 50 years, significant gaps have existed in gender and racial diversity across various medical specialties, despite the many benefits of a diverse physician workforce. One proposed approach to increasing diversity is top-down diversification, in which diverse leadership results in increased minority and female workforce representation.

**Objective:**

To investigate the changes in academic medical leadership diversity from 2007 to 2019 and to assess the recent leadership diversity of various specialties compared with the averages across all specialties.

**Design, Setting, and Participants:**

This was a cross-sectional analysis of physicians in varying academic roles in 2007, 2019, and 2020. Demographic data were collected via specialized reports from the Association of American Medical Colleges. Included were 4 primary care specialties (internal medicine, family medicine, pediatrics, obstetrics/gynecology [OB/GYN] and 4 surgical specialties (orthopedic surgery, neurologic surgery, otolaryngology [ENT], general surgery). Study participants were faculty, program directors, and chairpersons. Data were analyzed for the years 2007, 2019, and 2020.

**Intervention:**

Self-reporting of demographic information to residency programs collected via the Graduate Medical Education Track Survey.

**Main Outcomes and Measures:**

Proportions of each race/ethnicity and sex among cohorts of participants and comparisons between them.

**Results:**

The total number of individuals investigated included 186 210 faculty from 2019 (79 441 female [42.7%]), 6417 program directors from 2020 (2392 female [37.3%]), 1016 chairpersons from 2007 (89 female [8.8%]), and 2424 chairpersons from 2019 (435 female [17.9%]). When comparing chairperson diversity from 2007 to 2019, only internal medicine and general surgery experienced significant increases in minority (aggregate category used throughout the investigation to refer to anyone who self-identified as anything other than non-Hispanic White) representation (90% increase [11.7 percentage points, from 13.0% in 2007 to 24.7% in 2019]; *P* = .01 and 96% increase [13.0 percentage points, from 13.5% in 2007 to 26.5% in 2019]; *P* < .001), respectively; meanwhile, several specialties saw significant increases in female representation during this period (family medicine by 107.4%, *P* =.002; pediatrics by 83.1%, *P* =.006; OB/GYN by 53.2%, *P* =.045; orthopedic surgery by +4.1 percentage points, *P* =.04; general surgery by 226.9%, *P* =.005). In general, surgical specialties had lower leadership diversity than the average diversity of all residency programs, whereas primary care specialties had similar or increased diversity.

**Conclusions and Relevance:**

Study results suggest that some specialties have made significant contributions toward bridging diversity gaps whereas others continue to lag behind. Our recommendations to improve academic medical leadership diversity include programs and institutions (1) publishing efforts and outcomes of diversity representation, (2) incorporating a representative demographic for leadership selection committees, and (3) actively promoting the importance of diversity throughout the selection process.

## Key Points

**Question:**

What are the diversity demographics of academic medical leadership in various specialties compared with the average across all residency programs?

**Findings:**

In this cross-sectional study of academic medical leadership (105 044 faculty from 2019, 6131 program directors from 2020, 1016 chairpersons from 2007, and 2424 chairpersons from 2019), surgical specialties consistently had lower leadership diversity than the average across all residency programs, whereas primary care specialties typically had similar or increased diversity.

**Meaning:**

Select specialties in academic medicine have bridged diversity gaps in academic medical leadership whereas others continue to lag behind.

## Teaser

This cross-sectional study investigates the diversity demographics of academic medical leadership among various medical specialties compared with the average of all residency programs.

## Introduction

For more than 50 years, the medical community has focused on the importance of diversity throughout the profession.^1,2,3,4,5,6,7,8^ Some of the benefits of a more diverse physician population include higher patient-rated care, improved physician-patient communication, better preparation to treat marginalized racial and ethnic populations, and increased interest in working for underserved populations.^1,2,3,4,5,6,7,9,10,11,12,13,14^ Although efforts to improve diversity among medical students started in the 1970s, specialty-specific studies on diversity progression began to appear in the late 1980s and the 1990s.^3,15,16,17^ Starting in the early 1980s, several studies in various medical fields, including general surgery, dermatology, and family medicine, have revealed significant differences in gender and racial diversity within specialties.^4,9,17,18,19,20,21,22,23,24,25,26,27,28^ Furthermore, some studies have demonstrated sex disparities between surgical and nonsurgical specialties.^17,18,29^

A proposed approach to increasing diversity is “top-down” diversification, which refers to diverse leadership within a specialty resulting in higher levels of minority and female representation within the workforce.^30,31,32,33,34,35,36^ Although this strategy is relatively new in medicine, research in industries outside of health care, including financial investment, supermarket management, and professional librarians, has already demonstrated leadership teams’ ability to enhance the diversity of their underlying workforce.^31,32,33,34,35,36^ In addition to increased racial, ethnic, and gender representation within the workforce, a study^37^ conducted by the Boston Consulting Group found that companies with more diverse leadership teams had increased innovation revenue and greater financial performance. Recently, there have been pediatric, general surgery, and orthopedic surgery programs that have been successful with top-down diversification.^23,37,38^

Despite the demonstration of a lack of diversity over the last 40 years and numerous organizations, including the Association of American Medical Colleges (AAMC), speaking to this issue, progress has been relatively slow in several medical fields. Over the last 20 years, numerous recurrent studies within the fields of academic medicine, including general surgery, family medicine, and orthopedic surgery, have found the same lack of representation as that found in the 1980s when these studies began.^20,21,22,23,24,25,26,27,28,29,38,39,40,41,42^ Therefore, a different approach using diversity of leadership could be helpful in transforming academic medicine into a more diverse field. However, in order to use this top-down approach in medicine, one must first start with an understanding of the diversity of existing leaders in medicine and whether that has changed over time.

The primary purpose of our study was to assess the recent diversity of academic medical leadership in terms of program directors and chairpersons for several surgical and nonsurgical specialties in comparison with the averages across all specialties. Our secondary objective was to examine changes in chairperson diversity from 2007 to 2019 using a snapshot analysis for each specialty. For continuity, the specialties chosen have been similarly analyzed in other studies.^17,18,41^ Although diversity in medicine has been previously examined in residents and faculty members,^8,20,23,25,28,30,40,41,42^ few studies have included the representation of program directors and chairpersons.

## Methods

Faculty and leadership demographic data were collected from a nationally recognized source for the diversity of medicine: the AAMC. Data were obtained through specialized AAMC reports titled “Diversity in Medicine: Faculty Roster for 2007, 2019, and 2020.”^43,44,45,46^ Specialized AAMC data reports use self-reported demographic data from the Graduate Medical Education Track Survey sent to all residencies accredited by the Accreditation Council for Graduate Medical Education (ACGME). Institutional review board requirements were officially waived by an institutional review board ethics committee owing to the lack of direct human participants within the investigation. Using these reports, we analyzed the racial, ethnic, and gender compositions of faculty, program directors, and chairpersons in ACGME-accredited residency programs in the US for 4 primary care and 4 surgical fields. For purposes of continuity with other published studies comparing the diversity of medical specialties,^40,41,47,48^ we chose to analyze internal medicine, family medicine, pediatrics, and obstetrics/gynecology (OB/GYN) for the primary care specialties and orthopedic surgery, neurologic surgery, otolaryngology (ENT), and general surgery for the surgical specialties. This study followed the Strengthening the Reporting of Observational Studies in Epidemiology (STROBE) reporting guidelines.

Using categories from the AAMC’s Faculty Roster,^49^ the AAMC FACTS Glossary,^43^ and the US Census Bureau (USCB),^50^ race was defined as Asian American, Black or African American, White, or other, which included American Indian or Alaska Native, multiracial, Native Hawaiian or Pacific Islander, other, or unknown. According to the AAMC’s FACTS glossary^43^ and the USCB,^51^ ethnicity was separated into non-Hispanic or Latino and Hispanic or Latino. Based on demographic data from the USCB,^52^ a race or ethnicity must represent at least 5.0% of the general US population for individual representation in data analysis. To account for this, we combined the underrepresented groups falling below this threshold, including American Indian or Alaskan Native, multiracial, Native Hawaiian or Pacific Islander, other, or unknown, into a category titled “other.” Using the Population Reference Bureau’s definition of an individual identifying with a racial and ethnic group other than non-Hispanic White,^53^ a “minority” category was aggregated and used as a generalized measure of racial and ethnic diversity. Despite Asian Americans no longer being considered underrepresented in medicine by a majority of academic medical institutions, this race category was still considered under the broader minority category in order to represent them individually in our analysis, as is consistent with other studies on diversity in medicine.^1,7,14,31,32,38,41,48,54,55,56,57,58^ Thus, leadership diversity was defined as program directors and chairpersons identifying as female or any racial and ethnic group other than non-Hispanic White.

Because leadership roles are likely to be chosen from a recent composition of faculty members, our analysis compared faculty from 2019 with program directors in 2020 and chairpersons in 2019. Furthermore, we performed a snapshot comparison of minority and female chairpersons from 2007 to 2019. Lastly, we analyzed the diversity of program directors and chairpersons in each specialty by comparing their respective proportional racial and ethnic and gender compositions with the average leadership diversity metrics across all residency programs.

### Statistical Analysis

Statistical proportions were compared using 1- or 2-sample χ^2^ tests. If at least 25% of the sample count was less than 5, Fischer exact test was used. Statistical significance was set at a 2-sided *P* value < .05. All analyses were performed using SAS, version 9.4 (SAS Institute). Data were analyzed for the years 2007, 2019, and 2020.

## Results

### Diversity of Faculty (2019) Compared With 2019/2020 Leadership

The total number of individuals investigated included 186 210 faculty from 2019, 6417 program directors from 2020, 1016 chairpersons from 2007, and 2424 chairpersons from 2019. All together, 79 441 of the 186 210 faculty members in 2019 were female (42.7%) and self-identified with the following race and ethnicity groups: 6889 African American (4.2%), 38 100 Asian American (20.3%), 6197 Hispanic or Latino (3.6%), 117 914 White (63.3%), and 17 110 other (8.8%). A total of 2392 of 6417 program directors in 2020 were female (37.3%) and self-identified with the following race and ethnicity groups: 217 African American (3.5%), 1586 Asian American (25.9%), 306 Hispanic or Latino (5.0%), 3893 White (63.5%), and 129 other (2.1%). A total of 89 chairpersons in 2007 were female (8.8%) and self-identified with the following race and ethnicity groups: 53 African American (5.2%), 37 Asian American (3.6%), 35 Hispanic or Latino (3.4%), 861 White (84.7%), and 30 other (3.0%). A total of 435 chairpersons in 2019 were female (17.9%) and self-identified with the following race and ethnicity groups: 102 African American (4.2%), 261 Asian American (10.8%), 90 Hispanic or Latino (3.7%), 1885 White (77.8%), and 86 other (3.5%).

 When analyzing program director demographics in 2020 compared with that of faculty in 2019, program directors for 5 of 8 specialties demonstrated significantly lower minority representation than faculty (internal medicine, 834 [36.6%] vs 18 508 [41.4%]; *P* < .001; pediatrics, 287 [28.3%] vs 8470 [35.1%]; *P* < .001; orthopedic surgery, 80 [20.5%] vs 1090 [25.0%]; *P *= .047; neurologic surgery, 24 [23.3%] vs 753 [35.0%]; *P *= .01; general surgery, 105 [26.9%] vs 4800 [33.4%]; *P* = .001) (Table 1). Meanwhile, OB/GYN and ENT program director pools had similar minority representation (OB/GYN: 100 [31.1%]; *P* = .43; ENT: 41 [27.7%]; *P* = .42), and family medicine had significantly increased minority diversity in its program director pool (722 [55.4%] vs 2019 faculty, 2087 [33.4%]; *P* < .001) (Table 1). This was primarily due to the significantly greater proportion of Asian American representation, which was 12.3% (769 of 6249) of faculty in 2019 and 47.8% (623 of 1303; *P* <.001) among program directors in 2020. With the exception of ENT (18 [21.2%] vs faculty 2019, 750 [30.9%]; *P* = .06), chairpersons in all included specialties were significantly less racially and ethnically diverse than the respective faculty of each specialty due to their lower percentages of minority representation (internal medicine, 44 [24.7%] vs 18 508 [41.4%]; *P* < .001; family medicine, 35 [24.1%] vs 2087 [33.4%]; *P* =.02; pediatrics, 35 [22.7%] vs 8470 [35.1%]; *P* = .001; OB/GYN, 34 [21.8%] vs 2264 [33.8%]; *P* = .002; orthopedic surgery, 15 [12.3%] vs 1090 [25.0%]; *P* = .001; neurologic surgery, 21 [21.4%] vs 753 [35.0%]; *P* = .01; general surgery, 75 [26.5%] vs 4800 [33.4%]; *P* = .01) (Table 1). When comparing the proportions of each race and ethnicity in program directors and chairpersons with their respective proportions in faculty, almost every specialty (save for chairpersons in pediatrics and orthopedic surgery (pediatrics, 8 [5.2%] vs 2197 [9.1%]; *P* = .09; orthopedic surgery, 3 [2.5%] vs 305 [7.0%]; *P* = .05) demonstrated significantly lower diversity in the other category in both leadership positions (program directors [2020] vs faculty [2019]: internal medicine, 64 [2.8%] vs 3930 [8.8%]; *P* < .001; family medicine, 23 [1.8%] vs 644 [10.3%]; *P* < .001; pediatrics, 19 [1.9%] vs 2197 [9.1%]; *P* <.001; OB/GYN, 6 [1.9%] vs 535 [8.0%]; *P* < .001; orthopedic surgery, 5 [1.3%] vs 305 [7.0%]; *P* < .001; neurologic surgery, 0 [0%] vs 187 [8.7%]; *P* = .002; ENT, 1 [0.7%] vs 180 [7.4%]; *P* = .002; general surgery, 10 [1.8%] vs 1249 [8.7%]; *P* < .001; chairpersons [2019] vs faculty [2019]: internal medicine, 6 [3.4%] vs 3930 [8.8%]; *P* = .01; family medicine, 4 [2.8%] vs 644 [10.3%]; *P* = .003; OB/GYN, 5 [3.2%] vs 535 [8.0%]; *P* = .03; neurologic surgery, 2 [2.0%] vs 187 [8.7%]; *P* = .02; ENT, 1 [1.2%] vs 180 [7.4%]; *P* = .03; general surgery, 7 [2.5%] vs 1249 [8.7%]; *P* < .001). Furthermore, program directors in neurologic surgery (1 [12.6%] vs 449 [20.9%]; *P* = .04) and chairpersons in 5 of 8 specialties had significantly lower proportions of Asian American chairpersons than their respective faculty representation in 2019 (internal medicine, 22 [12.4%] vs 11388 [25.5%]; *P* < .001; family medicine, 8 [5.5%] vs 769 [12.3%]; *P* = .01; pediatrics, 11 [7.1%] vs 4274 [17.7%]; *P* < .001; OB/GYN, 11 [7.1%] vs 856 [12.8%]; *P* = .03; orthopedic surgery, 6 [4.9%] vs 586 [13.4%]; *P* = .006). Although 2 of 8 specialties had significantly less African American program director representation (family medicine, 35 [2.7%] vs 400 [6.4%]; *P* < .001; pediatrics, 26 [2.6%] vs 1014 [4.2%]; *P* = .01), orthopedic surgery program directors and family medicine chairpersons had significantly higher African American representation than faculty in 2019 (orthopedic surgery program directors, 18 [4.6%] vs 113 [2.6%]; *P* = .02; family medicine chairpersons, 17 [11.7%] vs 400 [6.4%]; *P* = .01). Regarding Hispanic or Latino representation, 3 of 8 specialties had significantly increased program director diversity (internal medicine, 135 [5.9%] vs 1563 [3.5%]; *P* < .001; pediatrics, 59 [5.8%] vs 966 [4.0%]; *P* = .004; neurologic surgery, 9 [8.7%] vs 56 [2.6%], *P* < .001), and family medicine had significantly lower program director diversity than faculty (family medicine, 40 [3.1%] vs 285 [4.4%]; *P* = .047).

**Table 1.** Diversity of Academic Faculty (2019) vs Leadership

Specialty	Minority representation, No. (%)					Female representation, No. (%)				
	Faculty (2019)	Program directors (2020)	*P* value	Chairpersons (2019)	*P* value	Faculty (2019)	Program directors (2020)	*P* value	Chairpersons (2019)	*P* value
Internal medicine	18 508 (41.4)	834 (36.6)^a^	<.001	44 (24.7)^a^	<.001	18 376 (41.1)	878 (33.7)^a^	<.001	30 (16.9)^a^	<.001
Family medicine	2087 (33.4)	722 (55.4)^b^	<.001	35 (24.1)^a^	.02	3321 (53.1)	343 (38.4)^a^	<.001	45 (31.0)^a^	<.001
Pediatrics	8470 (35.1)	287 (28.3)^a^	<.001	35 (22.7)^a^	.001	14377 (59.5)	661 (58.1)	.35	47 (30.5)^a^	<.001
OB/GYN	2264 (33.8)	100 (31.1)	.43	34 (21.8)^a^	.002	4386 (65.6)	239 (61.1)	.07	45 (28.8)^a^	<.001
Orthopedic surgery	1090 (25.0)	80 (20.5)^a^	.047	15 (12.3)^a^	.001	868 (19.9)	40 (9.0)^a^	<.001	5 (4.1)^a^	<.001
Neurologic surgery	753 (35.0)	24 (23.3)^a^	.01	21 (21.4)^a^	.01	475 (22.1)	11 (9.3)^a^	<.001	4 (4.1)^a^	<.001
Otolaryngology (ENT)	750 (30.9)	41 (27.7)	.42	18 (21.2)	.06	856 (35.2)	47 (27.6)^a^	.045	3 (3.5)^a^	<.001
General surgery	4800 (33.4)	105 (26.9)^a^	.001	75 (26.5)^a^	.01	3972 (27.7)	173 (26.3)	.43	24 (8.5)^a^	<.001

Abbreviation: OB/GYN, obstetrics/gynecology.

^a^ As compared with faculty in 2019, indicates a statistically significantly lower proportion among leadership.

^b^ As compared with faculty in 2019, indicates statistically significantly higher proportion among leadership.

Regarding sex, there were no significant differences in the proportions of female faculty and program directors for pediatrics, OB/GYN, and general surgery (Table 1). When comparing female program directors (2020) to female faculty (2019), internal medicine, family medicine, orthopedic surgery, neurologic surgery, and ENT had significantly lower gender diversity in program directors compared with faculty (internal medicine, 878 [33.7%] vs 18 355 [41.1%]; *P* < .001; family medicine, 343 [38.4%] vs 3318 [53.1%]; *P* < .001; orthopedic surgery, 40 [9.0%] vs 867 [19.9%]; *P* < .001; neurologic surgery, 11 [9.3%] vs 475 [22.1%]; *P* < .001; ENT, 47 [27.6%] vs 856 [35.2%]; *P* = .045) (Table 1). In terms of chairpersons, all 8 specialties had a lower female representation than their respective faculty pools ((internal medicine, 30 [16.9%] vs 18 355 [41.1%], *P* < .001; family medicine, 45 [31.0%] vs 3318; *P* < .001; pediatrics, 47 [30.5%] vs 14 368 [59.5%]; *P* < .001; OB/GYN, 45 [28.8%] vs 4389 [65.6%]; *P* < .001; orthopedic surgery, 5 [4.1%] vs 867 [19.9%]; *P* < .001; neurologic surgery, 4 [4.1%] vs 475 [22.1%]; *P* < .001; ENT, 3 [3.5%] vs 856 [35.2%]; *P* < .001; general surgery, 24 [8.5%] vs 3978 [27.7%]; *P* < .001) (Table 1).

When analyzing all specialties together, both program directors and chairpersons had significantly lower gender representation when compared with the combined faculty in 2019 (program directors, 2392 [37.3%] vs 79 441 [42.7%]; *P* < .001; chairpersons, 435 [17.9%] vs 79 441 [42.7%]; *P* < .001); meanwhile only chairpersons had significantly lower minority representation (minority chairpersons, 539 [22.2%] vs 68 296 [36.7%]; *P* < .001). When analyzing each race/ethnicity individually for program directors, the aggregated specialties had similar African American representation (227 [3.5%] vs 6889 [3.7%]; *P* = .50), significantly lower representation in the other category (135 [2.1%] vs 17110 [9.2%]; *P* < .001), and significantly higher Asian American and Hispanic or Latino representation compared with faculty in 2019 (Asian American program directors, 1660 [25.9%] vs 38100 [20.5%]; *P* < .001; Hispanic or Latino program directors, 320 [5.0%] vs 6197 [3.3%]; *P* < .001). For chairpersons, the 8 aggregated specialties had significantly lower Asian American and other representations (Asian American chairpersons, 2163 [10.8%] vs 38 100 [20.5%]; *P* < .001; other chairpersons, 86 [3.5%] vs 17 110 [9.2%]; *P* < .001), and similar African American and Hispanic or Latino representation (African American chairpersons, 102 [4.2%] vs 6889 [3.7%]; *P* = .19; Hispanic or Latino chairpersons, 90 [3.7%] vs 6197 [3.3%]; *P* = .29).

### Comparative Growth of Chairperson Diversity From 2007 to 2019

When analyzing the racial and ethnic growth of chairpersons from 2007 to 2019, 6 of 8 specialties experienced no significant changes in minority representation (Table 2). On the other hand, internal medicine and general surgery demonstrated a 90% increase (11.7 percentage points, from 13.0% in 2007 to 24.7% in 2019; *P* = .01) and 96% increase (13.0 percentage points, from 13.5% in 2007 to 26.5% in 2019; *P* < .001), respectively, in the proportion of minority chairpersons (Table 2). In regard to gender diversity, neurologic surgery, ENT, and internal medicine experienced no significant growth in the proportion of female chairpersons (Table 2). However, the other 5 specialties exhibited significant increases in female chairperson representation: family medicine by 107.4% (16.0 percentage points, from 15.0% to 31.0%; *P* = .002), pediatrics by 83.1% (13.9 percentage points, from 16.7% to 30.5%; *P* = .006), OB/GYN by 53.2% (10.0 percentage points, from 18.8% to 28.8%; *P* = .045), orthopedic surgery by +4.1 percentage points (from 0% to 4.1%; *P* = .04), and general surgery by 226.9% (+5.9 percentage points, from 2.6% to 8.5%; *P* = .005) (Table 2).

**Table 2.** Diversity of Chairpersons by Specialty (2007 vs 2019)

Specialty	Diversity of chairpersons by specialty (2007 vs 2019)							
	Sample sizes, No.		Minority, No. (%)			Female, No. (%)		
	2007	2019	2007	2019	*P* value	2007	2019	*P* value
Internal medicine	138	178	18 (13.0)	44 (24.7)	.01	15 (10.9)	30 (16.9)	.13
Family medicine	127	145	21 (16.5)	35 (24.1)	.12	19 (15.0)	45 (31.0)	.002
Pediatrics	126	154	24 (19.0)	35 (22.7)	.45	21 (16.7)	47 (30.5)	.006
OB/GYN	138	156	26 (18.8)	34(21.8)	.53	26 (18.8)	45 (28.8)	.045
Orthopedic surgery	102	122	11 (10.8)	15 (12.3)	.73	0 (0)	5 (4.1)	.04
Neurologic surgery	80	98	16 (20.0)	21 (21.4)	.82	1 (1.3)	4 (4.1)	.26
Otolaryngology (ENT)	76	85	8 (10.5)	18 (21.2)	.07	1 (1.3)	3 (3.5)	.37
General surgery	229	283	31 (13.5)	75 (26.5)	<.001	6 (2.6)	24 (8.5)	.005

Abbreviation: OB/GYN, obstetrics/gynecology.

### Leadership Diversity of Surgical and Nonsurgical Specialties (2019/2020)

**Figure 1.**
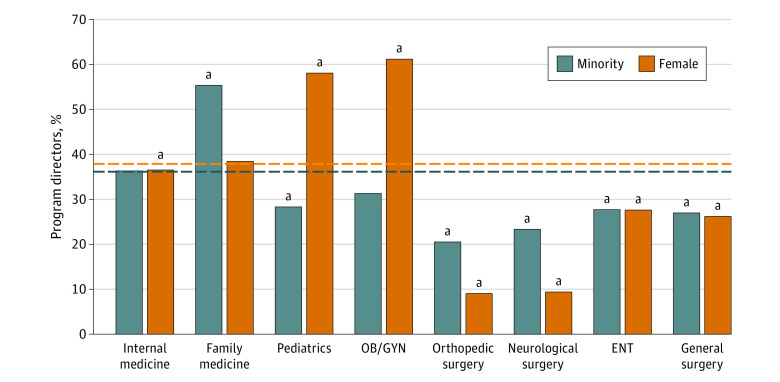
Minority and Female Program Directors by Specialty (2020) A bar graph illustrating the percentage of minority and female program directors for each specialty compared with the average percentage of minority or female program directors for all 8 specialties combined. Sample sizes are noted under each respective specialty. The dashed lines are visual representations of where the average (%) of minority or female representation lies for all specialties, which are the values to which the proportions of each individual specialties are compared. The *P* values for minority comparisons (from left to right) are as follows: *P* = .84, *P* < .001, *P* < .001, *P* = .052, *P* < .001, *P* = .01, *P* = .03, *P* < .001. The *P* values for female comparisons (from left to right) are as follows: *P* < .001, *P* = .50, *P* < .001, *P* < .001, *P* < .001, *P* < .001, *P* = .01, *P* < .001. ENT indicates otolaryngology; OB/GYN, obstetrics and gynecology. ^a^Indicates a significant difference between the proportion of minority or female program directors for the specialty and the average proportion.

For our program director analysis, the proportional racial, ethnic, and sex compositions of the 8 included specialties were combined into an aggregate “all specialties” category to compare each respective specialty with the average diversity of the included specialties. In the all specialties pool, 36.5% of the program directors (2238 of 6131) were a minority (Figure 1). On comparing this average percentage with each specialty, orthopedic surgery, neurologic surgery, ENT, general surgery, and pediatrics had significantly lower minority proportions (orthopedic surgery, 80 [20.5%] vs 2238 [36.5%]; *P* < .001; neurologic surgery, 24 [23.3%] vs 2238 [36.5%]; *P* = .01; ENT, 41 [27.7%] vs 2238 [36.5%]; *P* = .03; general surgery, 150 [26.9%] vs 2238 [36.5%]; *P* < .001; pediatrics, [28.3%] 287 vs 2238 [36.5%]; *P* < .001) (Figure 1). In contrast, family medicine had significantly greater diversity compared with the average minority proportion (family medicine, 1303 [55.4%] vs 2238 [36.5%]; *P* < .001) (Figure 1). When averaging each racial and ethnic category individually for all specialties, the average demographic distribution for program directors was 3.5% African American (217 of 6131), 25.9% Asian American (1586 of 6131), 5.0% Hispanic or Latino (306 of 6131), 63.5% White (3893 of 6131), and 2.1% other (129 of 6131) (Table 3). When compared with these averages, 5 of 8 specialties had significantly lower than average Asian American representation, whereas family medicine had significantly higher than average Asian American representation (orthopedic surgery, 44 [11.3%] vs 1586 [25.9%]; *P* < .001; neurologic surgery, 13 [12.6%] vs 1586 [25.9%]; *P* = .002; general surgery, 89 [16%] vs 1586 [25.9%]; *P* < .001; pediatrics, 183 [18%] vs 1586 [25.9%]; *P* < .001; OB/GYN, 51 [15.9%] vs 1586 [25.9%]; *P* < .001; family medicine, 623 [47.8%] vs 1586 [25.9%]; *P* < .001) (Table 3). Regarding African American representation, only OB/GYN showed a significantly higher than average proportion of African American program directors (29 [9.1%] vs 217 [3.5%]; *P* < .001) (Table 3). For Hispanic or Latino representation, family medicine had lower than average representation (41 [3.1%] vs 306 [5.0%]; *P* = .004) (Table 3). Meanwhile, only internal medicine had higher than average other representation (65 [2.8%] vs 129 [2.1%]; *P* = .047) (Table 3). In terms of female program directors, orthopedic surgery, neurologic surgery, ENT, general surgery, and internal medicine displayed significantly lower diversity when compared with the all-specialties average of 37.3% (orthopedic surgery, 40 [9%] vs 2392 [37.3%]; *P* < .001; neurologic surgery, 11 [9.3%] vs 2392 [37.3%]; *P* < .001; ENT, 47 [27.6%] vs 2392 [37.3%]; *P* = .01; general surgery, 173 [26.3%] vs 2392 [37.3%]; *P* < .001; internal medicine, 878 [33.7%] vs 2392 [37.3%]; *P* = .001) (Figure 1). In contrast, pediatrics and OB/GYN showed significantly higher than average gender diversity (pediatrics, 661 [58.1%] vs 2392 [37.3%]; *P* < .001; OB/GYN 239 [61.1%] vs 2392 [37.3%]; *P* < .001) (Figure 1).

**Table 3.** Diversity of Leadership by Specialty (2019/2020)

Leadership	No. (%)																
	Orthopedic surgery	*P* value	Neurologic surgery	*P* value	ENT	*P *value	General surgery	*P* value	Internal medicine	*P* value	Family medicine	*P* value	Pediatric medicine	*P* value	OB/GYN	*P* value	All specialties
**Program Directors (2020)**																	
Race and ethnicity, total No.	390	NA	103	NA	148	NA	557	NA	2295	NA	1303	NA	1015	NA	320	NA	6131
Minority^a^	80 (20.5)^b^	<.001	24 (23.3)^b^	.006	41 (27.7)^b^	.03	150 (26.9)^b^	<.001	834 (36.3)	.89	722 (55.4)^c^	<.001	287 (28.3)^b^	<.001	100 (31.3)	.06	2238 (36.5)
Asian American	44 (11.3)^b^	<.001	13 (12.6)^b^	.002	30 (20.3)	.12	89 (16.0)^b^	<.001	553 (24.1)	.10	623 (47.8)^c^	<.001	183 (18.0)^b^	<.001	51 (15.9)^b^	<.001	1586 (25.9)
Black	18 (4.6)	.27	2 (1.9)	.38	2 (1.4)	.15	24 (4.3)	.35	81 (3.5)	.98	35 (2.7)	.12	26 (2.6)	.11	29 (9.1)^c^	<.001	217 (3.5)
Hispanic or Latino	13 (3.3)	.14	9 (8.7)	.09	8 (5.4)	.82	27 (4.8)	.88	135 (5.9)	.10	41 (3.1)^b^	.004	59 (5.8)	.27	14 (4.4)	.62	306 (5.0)
Other^d^	5 (1.3)	.27	0 (0)	.14	1 (0.7)	.23	10 (1.8)	.62	65 (2.8 )^c^	.047	23 (1.8)	.43	19 (1.9)	.63	6 (1.9)	.78	129 (2.1)
Sex, total No.	444	NA	118	NA	170	NA	659	NA	2605	NA	893	NA	1137	NA	391	NA	6417
Female	40 (9.0)^b^	<.001	11 (9.3)^b^	<.001	47 (27.6)^b^	.01	173 (26.3)^b^	<.001	878 (33.7)^b^	.001	343 (38.4)	.51	661 (58.1)^c^	<.001	239 (61.1)^c^	<.001	2392 (37.3)
**Chairpersons (2019)**																	
Total No.	122	NA	98	NA	85	NA	283	NA	178	NA	145	NA	154	NA	156	NA	2424
Minority	15 (12.3)^b^	.009	21 (21.4)	.85	18 (21.2)	.82	75 (26.5)	.10	44 (24.7)	.44	35 (24.1)	.59	35 (22.7)	.89	34 (21.8)	.90	539 (22.2)
Asian American	6 (4.9)^b^	.04	14 (14.3)	.27	12 (14.1)	.33	41 (14.5)^c^	.06	22 (12.4)	.51	8 (5.5)^b^	.045	11 (7.1)	.16	11 (7.1)	.14	261 (10.8)
Black	4 (3.3)	.62	1 (1.0)	.12	1 (1.2)	.17	14 (4.9)	.56	8 (4.5)	.85	17 (11.7)^c^	<.001	8 (5.2)	.56	10 (6.4)	.19	102 (4.2)
Hispanic or Latino	2 (1.6)	.23	4 (4.1)	.85	4 (4.7)	.64	13 (4.6)	.46	8 (4.5)	.60	6 (4.1)	.79	8 (5.2)	.35	8 (5.1)	.37	90 (3.7)
Other	3 (2.5)	.13	2 (2.0)	.92	1 (1.2)	.27	7 (2.5)	.11	6 (3.4)	.14	4 (2.8)	.81	8 (5.2)^c^	.006	5 (3.2)	.67	86 (3.5)
Female	5 (4.1)^b^	<.001	4 (4.1)^b^	<.001	3 (3.5)^b^	<.001	24 (8.5)^b^	<.001	30 (16.9)	.71	45 (31.0)^c^	<.001	47 (30.5)^c^	<.001	45 (28.8)^c^	<.001	435 (17.9)

Abbreviations: ENT, otolaryngology; NA, not applicable; OB/GYN, obstetrics/gynecology.

^a^ Using the Population Reference Bureau’s definition of an individual identifying with a racial and ethnic group other than non-Hispanic White, a minority category was aggregated and used as a generalized measure of racial and ethnic diversity.

^b^ Indicates a statistically significantly lower proportion among leadership.

^c^ Indicates statistically significantly higher proportion among leadership.

^d^ The other category includes American Indian or Alaska Native, multiracial, Native Hawaiian or Pacific Islander, other, and unknown.

**Figure 2.**
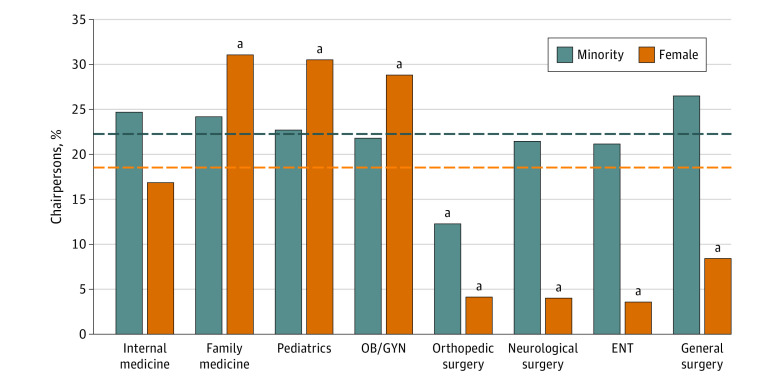
Minority and Female Chairpersons by Specialty (2019) A bar graph illustrating the percentage of minority and female chairpersons for each specialty compared with the average percentage of minority or female chairpersons for all residency programs combined. Sample sizes are noted at the bottom of the bars. The dashed lines are visual representations of where the average (%) of minority or female representation lies for all specialties, which are the values to which the proportions of each individual specialties are compared. The *P* values for minority comparisons (from left to right) are as follows: *P* = .43, *P* = .59, *P* = .98, *P* = .90, *P* = .008, *P* = .92, *P* = .82, *P* = .08. The *P* values for female comparisons (from left to right) are as follows: *P* = .72, *P* < .001, *P* < .001, *P* < .001, *P* < .001, *P* < .001, *P* < .001, *P* < .001. ENT indicates otolaryngology; OB/GYN, obstetrics and gynecology. ^a^Indicates a significant difference between the proportion of minority or female chairpersons for the specialty and the average proportion across all programs.

When analyzing the average diversity demographics of chairpersons across all programs, the average proportion of minority chairpersons was 22.2% (539 of 2424) (Figure 2). Although 7 of 8 included specialties showed no significant differences in their proportions of minority chairpersons when compared with the average, orthopedic surgery was the only specialty found to have significantly lower minority diversity at 12.3% (15 [12.3%] vs 538 [22.2%]; *P* = .008) (Figure 2). When averaging each racial and ethnic category individually across all programs, the demographic distribution for chairpersons was 4.2% African American (102 of 2424), 10.8% Asian American (261 of 2424), 3.7% Hispanic or Latino (90 of 2424), 77.8% White (1885 of 2424), and 3.5% other (86 of 2424). When compared with these averages, 2 of 8 specialties had significantly lower than average Asian American diversity (family medicine, 8 [5.5%] vs 262 [10.8%]; *P* = .045; orthopedic surgery, 6 [4.9%] vs 262 [10.8%]; *P* = .04), whereas general surgery had significantly higher than average Asian American diversity (41 [14.5%] vs 262 [10.8%]; *P* < .05) (Table 3). Meanwhile, family medicine had significantly higher African American representation than the average (17 [11.7%] vs 102 [4.2%]; *P* < .001). All 8 specialties had similar proportions of Hispanic or Latino chairpersons compared with the average. For other representation, pediatrics had greater representation compared with the average (12 [7.9%] vs 85 [3.5%]; *P* = .006). In regard to female chairpersons, orthopedic surgery, neurologic surgery, ENT, and general surgery displayed significantly lower than average gender diversity when compared with the average of all specialties together at 17.9% (435 of 2424; orthopedic surgery, 5 [4.1%] vs 434 [17.9%]; *P* < .001; neurologic surgery, 4 [4.1%] vs 434 [17.9%]; *P* < .001; ENT, 3 [3.5%] vs 434 [17.9%]; *P* < .001; general surgery, 24 [8.5%] vs 434 [17.9%]; *P* < .001) (Figure 2). In contrast, family medicine, pediatrics, and OB/GYN had significantly higher than average female diversity (family medicine, 45 [31.0%] vs 434 [17.9%]; *P* < .001; pediatrics, 47 [30.5%] vs 434 [17.9%]; *P* < .001; OB/GYN, 45 [28.8%] vs 434 [17.9%]; *P* < .001) (Figure 2).

## Discussion

Although diversification of academic medical leadership has been proposed as a potential solution to the lack of minority and female representation in medicine, there have been limited studies exploring this topic. Results of our cross-sectional analysis suggest that when compared with faculty in 2019, only program directors in family medicine had greater minority representation than their faculty counterpart, which was primarily caused by the greater proportion of Asian Americans. However, the majority of chairperson and program director analyses of minority and female representation for each specialty showed decreased diversity when compared with faculty in 2019, specifically in the categories of Asian American and other. Although some specialties demonstrated significant increases in minority or female chairperson representation from 2007 to 2019, all specialties displayed significantly lower racial, ethnic, and gender diversity among chairpersons in 2019 when compared with faculty in 2019, with the exception of ENT. These data suggest that, although there have been some gains across various specialties in terms of leadership diversification, there is still a decrease in minority and gender diversity when advancing from faculty to leadership positions in academic medicine.

A key finding of our study was that surgical specialties had consistently lower leadership diversity when compared with the average diversity of all residency programs, whereas primary care specialties typically had similar or increased diversity. All 4 surgical specialties had significantly lower proportions of minority program directors, female program directors, and female chairpersons than the average for all specialties. However, in terms of minority chairperson diversity, 3 of 4 surgical specialties showed similar diversity to the average across all specialties. In contrast, orthopedic surgery was the only specialty with significantly lower minority chairperson representation than the average. Our results suggest that surgical specialties lag in leadership diversity, which is similar to previous studies that have shown a gap between surgical specialties and primary care specialties in terms of resident and faculty diversity.^17,18,29^

Studies examining a top-down approach to diversification have now begun to highlight the correlation between diverse faculty and increased diversity in residents.^23,30,37,38,42^ In 2 studies,^30,42^ one investigating gender diversity in orthopedic applicants at 107 medical schools and the other investigating residents in 143 orthopedic residency programs, increased gender diversity among both applicants and residents was either influenced by (odds ratio, 1.3; *P* = .02) or connected to (triple the number of female leadership; *P* < .001) with increased female orthopedic faculty and women in leadership positions. With regard to racial diversity, a 2020 study^38^ out of the Children’s Mercy Kansas City (CMKC) pediatrics program found that with the addition of minority faculty during the residency selection process and a diversity committee, their 2019 residency class was the most diverse class in recent history. In addition, more minority residents elected to remain at the CMKC for their fellowship programs.^38^ Based on these findings that show a strong influence of diverse faculty on residents and applicants, the same concept may apply to diversity in chairpersons who typically have the opportunity to build leadership teams of residency program directors and faculty. Based on our findings, supporting research, and initiatives by the AAMC,^59^ we recommend 3 possible solutions to help bridge the diversity gaps in academic medical leadership: (1) advocating for programs and institutions to proactively publish the efforts and outcomes of diversity representation, (2) incorporating a representative proportion of minority and female members on academic medicine leadership selection committees, and as has been effective in other industries,^31,32,33,34,35,36^ (3) encouraging leaders of academic health care systems to actively promote the importance of leadership diversity to their selection committee members.

### Limitations

Our study has several limitations. First, the data used in this study included self-reported race, ethnicity, and sex. This method allows for self-report bias and potential errors in self-reporting. Second, respondents to the Graduate Medical Education Track surveys used to collect data for the AAMC database were not prohibited from selecting multiple races or ethnicities from which they identified. Therefore, for statistical analysis, we used the sum of responses for each separate race or ethnicity selection as the total sample size instead of the total sample size reported by the AAMC. Additionally, the sample size of chairpersons was smaller relative to the other investigated populations; this limited our statistical analysis, and therefore, this may have underestimated statistical significance.

## Conclusions

In this cross-sectional study, results suggest that the majority of specialties in academic medicine saw no improvement in racial diversity despite the greater diversity of the pool of available faculty members to choose from; meanwhile, several specialties did experience significant increases in gender diversity among their leadership. However, all of the specialties analyzed continued to have lower leadership diversity when compared with their own faculty members, with many of the surgical specialties having lower than average racial and gender diversity in their leadership. In conclusion, persistent monitoring of diversity in leadership allows us to accurately assess the trends of diversity in medicine and determine the potential to promote a diverse physician workforce.

## Notes

Supplement.
Data Sharing Statement
