# Supplementary material for: Racial, Ethnic, and Sex Diversity in Academic Medical Leadership
Source: JAMA Netw Open. 2023 Sep 25;6(9):e2335529. doi: 10.1001/jamanetworkopen.2023.35529 (PMC10520740; doi:10.1001/jamanetworkopen.2023.35529)
Supplement: Supplement. — Data Sharing Statement [file jamanetwopen-e2335529-s001.pdf]

Data Sharing Statement

Meadows. Racial, Ethnic, and Sex Diversity in Academic Medical Leadership. *JAMA Netw Open*. Published September 25, 2023. doi:10.1001/jamanetworkopen.2023.35529

Data
Data available: No

Additional Information
Explanation for why data not available: Authors have elected not to share data from this investigation at this time due to agreements made to the AAMC where specialized data reports were obtained.
